# Desire for social distance towards mentally ill people

**DOI:** 10.1192/j.eurpsy.2025.1583

**Published:** 2025-08-26

**Authors:** K. Evangelia, A. Argyriadis, S. Kotrotsiou, E. Asimakopoulou

**Affiliations:** 1 Nursing, Frederick University, Nicosia, Cyprus; 2 Nursing, University of Patras, Patra, Greece

## Abstract

**Introduction:**

Familiarity with, social distance from people with mental illness and socio - demographic characteristics has been linked to conditions that lead to weaker prejudiced beliefs and a more positive opinion about mental illness.

**Objectives:**

The relation of the attitudes and perceptions of the citizens of Larissa city towards mental illness and their desire for social distance from the mentally ill people, in relation to their familiarity with mental illness and their demographic characteristics.

**Methods:**

A **convenience sampling survey** was conducted in Greece, **Larissa City (n=220).** The research tools which were used were: a) **Familiarity** was assessed using **the Level of Contact Report**, b) **The Social Distance Scale**, to measure the desire for social distance from people with mental illness c) Attitudes about mental illness were assessed using **The Opinions About Mental Illness (OMI) Questionnaire** and finally d) **a questionnaire for the socio - demographic information.**

**Results:**

Univariate and multivariate analysis was applied for the statistical analysis of the data, which showed that: Desire for social distance from people with mental illness is positively related with the familiarity (with mental illness) but on the other hand is negatively related to the opinions / attitudes about mental illness.

**Conclusions:**

The results from this study highlight and emphasize the important role of familiarity (with mental illness) and the desire for social distance (from people with mental illness) in the formation of positive/negative (prejudiced) attitudes and perceptions about mental illness.

**Disclosure of Interest:**

None Declared

